# An anti-CRF antibody suppresses the HPA axis and reverses stress-induced phenotypes

**DOI:** 10.1084/jem.20190430

**Published:** 2019-08-29

**Authors:** Hunter S. Futch, Karen N. McFarland, Brenda D. Moore, M. Zino Kuhn, Benoit I. Giasson, Thomas B. Ladd, Karen A. Scott, Melanie R. Shapiro, Rachel L. Nosacka, Marshall S. Goodwin, Yong Ran, Pedro E. Cruz, Daniel H. Ryu, Cara L. Croft, Yona Levites, Christopher Janus, Paramita Chakrabarty, Andrew R. Judge, Todd M. Brusko, Annette D. de Kloet, Eric G. Krause, Todd E. Golde

**Affiliations:** 1 McKnight Brain Institute, Center for Translational Research in Neurodegenerative Disease, Department of Neuroscience and Neurology, College of Medicine, University of Florida, Gainesville, FL; 2 McKnight Brain Institute, Department of Pharmacodynamics, College of Pharmacy, University of Florida, Gainesville, FL; 3 Diabetes Institute, Department of Pathology, Immunology and Laboratory Medicine, College of Medicine, University of Florida, Gainesville, FL; 4 Department of Physical Therapy, College of Public Health and Health Professions, University of Florida, Gainesville, FL; 5 McKnight Brain Institute, Department of Physiology and Functional Genomics, College of Medicine, University of Florida, Gainesville, FL

## Abstract

Hypothalamic–pituitary–adrenal (HPA) axis dysfunction contributes to numerous human diseases and disorders. We developed a high-affinity monoclonal antibody, CTRND05, targeting corticotropin-releasing factor (CRF). In mice, CTRND05 blocks stress-induced corticosterone increases, counteracts effects of chronic variable stress, and induces other phenotypes consistent with suppression of the HPA axis. CTRND05 induces skeletal muscle hypertrophy and increases lean body mass, effects not previously reported with small-molecule HPA-targeting pharmacologic agents. Multiorgan transcriptomics demonstrates broad HPA axis target engagement through altering levels of known HPA-responsive transcripts such as Fkbp5 and Myostatin and reveals novel HPA-responsive pathways such as the Apelin-Apelin receptor system. These studies demonstrate the therapeutic potential of CTRND05 as a suppressor of the HPA axis and serve as an exemplar of a potentially broader approach to target neuropeptides with immunotherapies, as both pharmacologic tools and novel therapeutics.

## Abstract (precis)

A high-affinity monoclonal antibody (CTRND05) targeting corticotropin-releasing factor (CRF) blocks stress-induced corticosterone increases, counteracts effects of chronic variable stress, and induces other phenotypes consistent with suppression of the HPA axis.

## Introduction

Epidemiological and biomarker studies have associated both early-life or long-term psychological stress as well as alterations in cortisol levels with numerous diseases and conditions (Sapolsky, 2000; de Kloet et al., 2005; Chrousos, 2009; Lupien et al., 2009; Incollingo Rodriguez et al., 2015; Gray et al., 2017; Zorn et al., 2017; Fogelman and Canli, 2018; Song et al., 2018). These studies, along with a wealth of experimental data, implicate the stress-responsive corticotropin-releasing factor (CRF; gene name *Crh*) and glucocorticoid (GC) signaling systems as potential targets in neuropsychiatric, cardiovascular, metabolic, and age-related degenerative conditions (Rosmond, 2005; Kehne and Cain, 2010; Sanders and Nemeroff, 2016; Futch et al., 2017; Spierling and Zorrilla, 2017; Soria et al., 2018).

The CRF family of neuropeptides are key orchestrators of the central response to psychological stress and include the urocortin (UCN) peptides in addition to CRF. The three urocortins, UCN1 (gene name *Ucn*), UCN2 (gene name *Ucn2*), and UCN3 (gene name *Ucn3*) share 32–43% homology to the 41-residue CRF neuropeptide (Dautzenberg and Hauger, 2002). CRF and the UCNs bind and activate the G protein–coupled receptors, CRFR1 and CRFR2 (gene names *Crhr1* and *Crhr2)* to varying degrees (Dautzenberg and Hauger, 2002); however, only CRF and UCN1 bind with high-affinity to CRFR1, whereas all UCNs bind CRFR2 with high affinity. CRFR1 acts within the central nervous system (CNS) in a nuclei-dependent fashion to augment stress and anxiety-related phenotypes (Dautzenberg and Hauger, 2002). As opposed to CRFR1, the role of the CRFR2 receptor is not well understood. Further, CRFR2 is expressed in discrete areas of the brain and is widely expressed in the periphery, as opposed to CRFR1, which is more broadly expressed throughout the brain. Activation of CRFR2 has been postulated to have various effects in the nervous, cardiovascular, intestinal, and skeletal muscle systems and can act to oppose the effects of CRFR1 receptor activation (Hauger et al., 2006). CRF activity can be regulated by its binding to the CRF binding protein (CRFBP, gene name *Crhbp*). High-affinity binding of CRF to CRFBP (K_d_ of 2.0 × 10^−10^) can block receptor engagement and decrease CRFR1 activation (Potter et al., 1991, 1992; Ketchesin et al., 2017). The CRFBP is mainly expressed in the brain of rodents and in the brain, liver, and placenta of primates.

CRF is the initiating factor of hypothalamic–pituitary–adrenal (HPA) axis activation. In response to psychological stress, CRF, released from the paraventricular nucleus of the hypothalamus, binds CRF receptors (CRFR1) in the anterior pituitary, where it stimulates release of adrenocorticotropic hormone (ACTH). ACTH binds melanocortin 2 receptors in the adrenal glands, stimulating production and release of GCs, namely cortisol in humans and corticosterone in rodents (Spencer et al., 2018). GCs bind to ubiquitous mineralocorticoid receptors and GC receptors (GRs), resulting in physiological adaptations that prepare the body to overcome ongoing or imminent stressors. GR activation is followed by sequestration of the receptor by its chaperone FKBP5, which prevents excess GR signaling (Zannas et al., 2016). Responses to GR signaling include increased vigilance, mobilization of energy stores, and vascular sympathetic reactivity. Chronic excess of these responses has been associated with increased anxiety and mood disturbance, insulin resistance, hypertension, and muscle and brain atrophy (Höschl and Hajek, 2001; Braun and Marks, 2015; Gueugneau et al., 2018). Cushing’s syndrome, a state of extreme GC excess, displays all of these features, many of which are normalized when GC levels are reduced (Starkman et al., 1999). Additionally, high cortisol has been associated with Alzheimer’s disease, major depression, cognitive decline in aging, and other disorders (Roy et al., 2012; Ennis et al., 2017; Futch et al., 2017; Zorn et al., 2017; Echouffo-Tcheugui et al., 2018; Matosin et al., 2018).

Small-molecule approaches to suppress the HPA axis have had limited therapeutic utility. GR antagonists suffer from lack of receptor specificity, and steroidogenesis inhibitors result in buildup of bioactive precursors; thus, both of these classes of drugs have dose-limiting side effects (Cadepond et al., 1997; Fleseriu and Petersenn, 2015). Further, as these drugs target the HPA axis at the GC or GR level, they would not be predicted to be optimal agents for conditions such as Alzheimer’s disease or irritable bowel syndrome where dysfunctional CRF signaling is implicated. There have also been intensive efforts to develop CRFR1 antagonists, but human studies with several of these drugs showed limited evidence for target engagement at the level of GC suppression (Spierling and Zorrilla, 2017).

**Figure 1.**
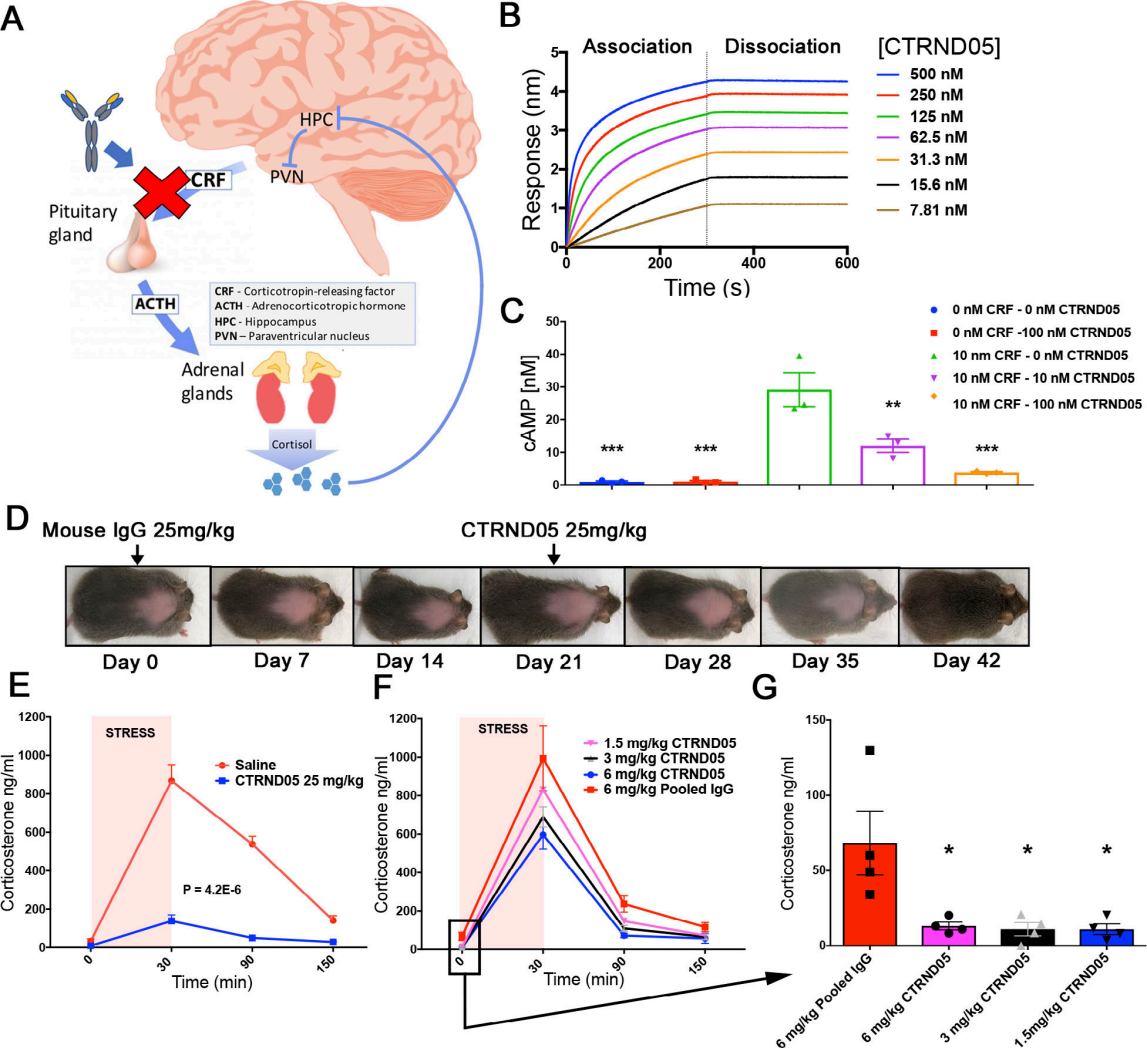
**High-affinity CTRND05 effectively engages target and blocks HPA axis activation. (A)** HPA axis schematic and the immunotherapeutic approach. **(B)** CTRND05 monoclonal antibody binding affinity for CRF detected by BLI with K_d_ < 1.0 × 10^−12^ (representative figure from three independent experiments). **(C)** H4 cells stably overexpressing CRFR1 treated with 10 nM CRF increase cyclic AMP production, and cotreatment with increasing concentrations of CTRND05 blocks this effect (*n* = 3 wells; data from one independent experiment). **(D)** B6C3H-F1 NTg mice transduced intracerebroventricularly at P0 with rAAV(2/8) CRF develop a Cushingoid phenotype. A single i.p. dose of CTRND05 at 25 mg/kg reverses skin pigmentation and hair loss over 3 wk, while injection of mouse IgG1 has no effect (replicates in Fig. S1 L, *n* = 4, 2 male/2 female, from two independent experiments). **(E)** NTg B6C3H-F1 mice (*n* = 10, 5 male/5 female from one independent experiment) were injected i.p. with CTRND05 (25 mg/kg) or saline, and after 16 h were exposed to 30 min of restraint stress. CTRND05 reduces the stress-induced increase in corticosterone levels. **(F and G)** NTg B6C3H-F1 mice (*n* = 4, 2 male/2 female from one independent experiment) were singly housed for 2 mo (mild stressor) and then treated with varying doses of CTRND05 and exposed to 30 min of restraint stress. CTRND05 reduced corticosterone response in a dose-dependent fashion and lowers baseline corticosterone level at low doses. Error bars are represented as mean ± SEM. Statistics: one-way ANOVA followed by Dunnett’s multiple comparison test (C and G); two-way ANOVA (E); *, P < 0.05; **, P < 0.01; ***, P < 0.001.

As an alternative to small-molecule therapeutics targeting the HPA axis, we have evaluated the potential of targeting CRF using a biological approach. CRF is present at low picomolar concentrations in both blood and cerebrospinal fluid and has a short half-life, making it an attractive target for passive immunotherapy (Fig. 1 A). Directly targeting CRF also offers advantages such as simultaneously blocking both HPA axis activation and GC-independent effects of CRF on immune, gut, and brain function (Lemos et al., 2012; Chatoo et al., 2018). Herein, we report on the development of a mouse monoclonal antibody, CTRND05, that binds CRF with high affinity (∼1 pM K_d_) and dose-dependently suppresses HPA axis activation in mice following peripheral administration. Additionally, CTRND05 administration was found to induce skeletal muscle hypertrophy and increase lean body mass. Multiorgan transcriptomics reveal widespread changes in all organs tested and identify novel HPA-responsive pathways such as the Apelin-Apelin receptor system.

## Results and discussion

We immunized mice with CRF peptides and isolated monoclonal anti-CRF antibodies following fusion of the splenocytes from mice with high-titer anti-CRF antibody responses (Fig. S1, A–C). Screening the resultant hybridomas enabled us to identify a murine IgG1 monoclonal antibody (CTRND05) that binds CRF with a high affinity (∼1 pM K_d_) as determined by biolayer interferometry (BLI; Fig. 1 B). CTRND05 blocked cAMP production in CRFR1-overexpressing H4 cells upon cotreatment of antibody and CRF (Fig. 1 C). I.p. injection of CTRND05 (25 mg/kg) 16 h before 30 min of restraint stress blocked acute increases in plasma corticosterone levels by ∼85% (Fig. 1 E). Neither vaccination of mice with the CRF-OVA immunogen (used to generate CTRND05; Fig. S1 D) nor passive immunization with a lower-affinity (∼2.0 × 10^−8^ K_d_) CRF antibody (CTRND01) blocked restraint stress–induced increases in plasma corticosterone levels (Fig. S1, E and F). Further characterization revealed that CTRND05 has a half-life of ∼1 wk in mice (Fig. S1 G). As previously reported, we observed that female mice (Fig. S1 H) had an increased corticosterone response compared with males (Fig. S1 I; Jones et al., 1998). Plasma corticosterone levels assessed on day 5 after 25 mg/kg i.p. injection demonstrated persistent HPA axis suppression (Fig. S1 J). Recombinant adeno-associated virus (rAAV)–mediated overexpression of CRF (Fig. S1 K; Chakrabarty et al., 2013) leads to a cushingoid phenotype in mice, similar to that seen in CRF-overexpressing transgenic mice (Wang et al., 2011), and a single 25 mg/kg i.p. injection of CTRND05 reversed the hair loss seen in these mice (Figs. 1 D and S1 L). CTRND05 displayed no cross reactivity to UCN1 and UCN3 when binding was assessed by direct ELISA, and minimal reactivity to 10 µM UCN2 (Fig. S1, M and N). We then evaluated the ability of UCNs to compete with CRF for CTRND05 binding via direct competitive ELISA. We observed that UCN1 demonstrated a competitive effect at higher concentrations (100 nM and 1 µM), and that UCN2 and UCN3 demonstrated no competitive effect (Fig. S1, O–Q). We determined the affinity of CTRND05 for UCNs using BLI under conditions identical to those used to determine binding of CTRND05 to CRF. These assays showed the following affinities: CRF, K_d_ < 1.0 × 10^−12^ (Fig. S1 S); UCN1, K_d_ = 4.26 × 10^−9^ (Fig. S1 T); UCN2, K_d_ = 4.0 × 10^−9^ (Fig. S1 U); and UCN3, K_d_ = 2.36 × 10^−9^ (Fig. S1 V). Therefore, the affinities as assessed by BLI of CTRND05 for UCNs are >4,000-fold lower than the affinity of CTRND05 for CRF.

Nontransgenic (NTg) B6C3H-F1 mice singly housed for 2 mo (mild stressor) and then injected i.p. with varying doses of CTRND05 16 h before restraint stress demonstrated a dose-dependent reduction in stress-induced plasma corticosterone levels; however, even the lowest dose (1.25 mg/kg) showed suppression of basal plasma corticosterone levels (Fig. 1, F and G). These data demonstrate that high-dose CTRND05 can block stress-induced GCs, whereas lower doses only partially block stress-induced GCs but suppress basal GC levels.

**Figure 2.**
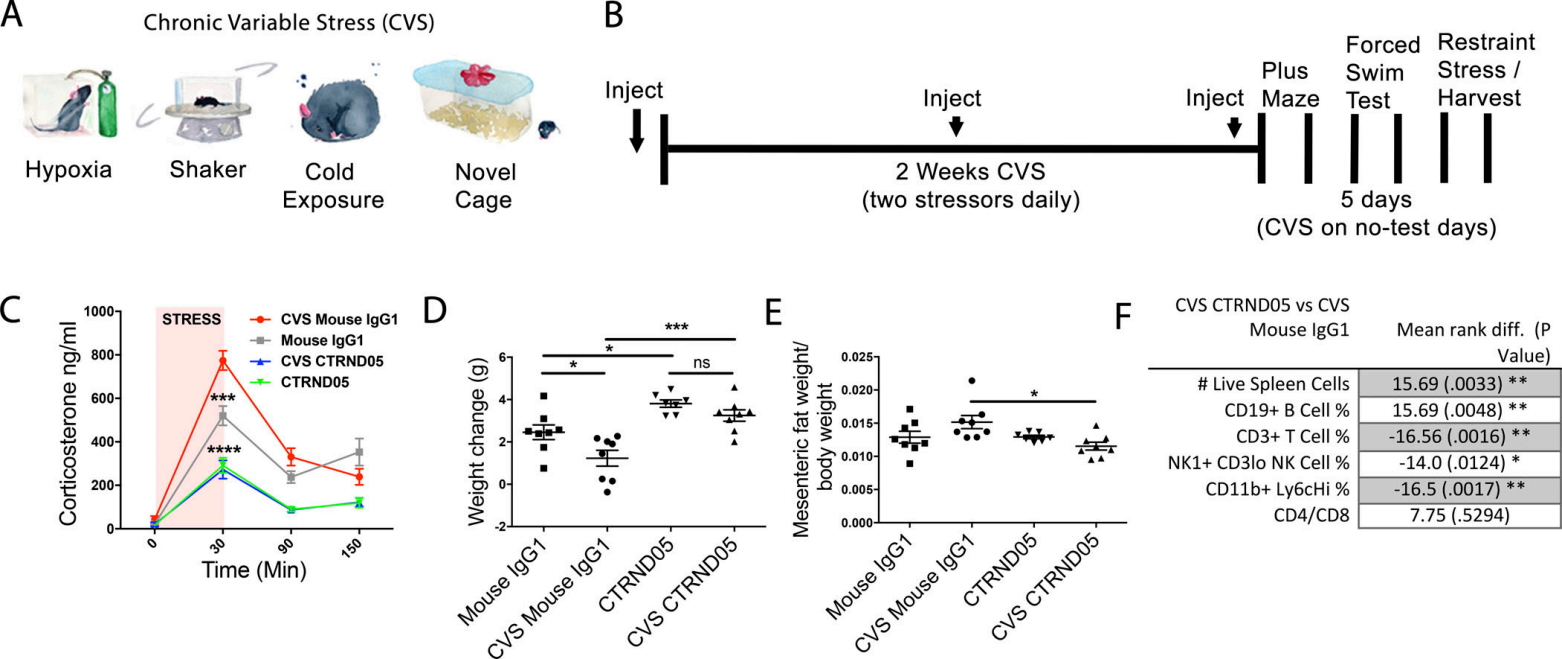
**CTRND05 blocks effects of CVS. (A–F)** C57BL/6J NTg mice (*n* = 8, 4 male/4 female each group except CTRND05 [*n* = 7, 4 male/3 female], from one independent experiment) were individually housed and i.p. injected with 25 mg/kg initially and then weekly with 12.5 mg/kg of CTRND05 or control mouse IgG1. **(A)** Stressors used in the CVS paradigm. **(B)** Experimental timeline. **(C)** CVS increases corticosterone response to a novel stressor (30-min restraint), and CTRND05 treatment blocks this effect (statistical comparison on 30-min time point). **(D)** CVS/mouse-IgG1 treated mice gain significantly less weight, an effect blocked by CTRND05. **(E)** CTRND05 administration significantly decreased mesenteric fat/body weight in CVS-treated mice, but not in non–CVS-treated mice. **(F)** CTRND05 treatment increases the number of live cells in the spleen and alters the distribution of immune cell populations in CVS/CTRND05-treated versus CVS/mouse IgG1–treated groups; plots for percentages/counts available on request. Error bars are represented as mean ± SEM. Statistics: one-way ANOVA followed by Dunnett’s multiple comparisons test (C–E); Dunn’s multiple comparison (F); *, P < 0.05; **, P < 0.01; ***, P < 0.001; ns, not significant.

To investigate whether CTRND05 could ameliorate chronic stress–induced phenotypes, sex-balanced groups of singly housed NTg C57BL/6J mice underwent either 2 wk of chronic variable stress (CVS; Fig. 2, A and B) or no additional stress and treatment with CTRND05 or a mouse IgG1 monoclonal control. Following the CVS paradigm, exposure to 30 min of restraint revealed that the CVS-mouse IgG1 group had a sensitized corticosterone response to this novel stressor, and both CTRND05 treatment groups experienced a significant reduction in their responses 5 d following the last dose (Fig. 2 C). Treatment with CTRND05 prevented the decrease in body weight gain seen in the CVS control group, and both CVS and non-CVS groups treated with CTRND05 experienced a significant increase in weight (Fig. 2 D). At study endpoint, we assessed the weight of GC-sensitive organs in addition to alterations in immune cell populations one would anticipate to observe with HPA axis blockade. Adrenal weights of CTRND05-treated mice were decreased, and thymus and spleen weights were increased, data congruent with GC suppression (Fig. S2, A–J). CVS-exposed mice treated with CTRND05 had reduced mesenteric fat (Fig. 2 E), despite having overall increased body weight. Analysis of splenic immune cell populations revealed significant increases in the number of live splenocytes, with an increase in B cell percentage and decrease in T cell percentage, increases in the absolute number of both B and T cells, but no significant change in the CD4/CD8 ratio (Fig. 2 F). Percentages of natural killer cells and inflammatory monocytes were also significantly reduced (Fig. 2 F). These shifts indicate that CTRND05 causes opposing effects on immune cell populations compared with those reported to be induced by stress paradigms (Li et al., 2018). Collectively, these data demonstrate suppression of GC release by CTRND05, and that CTRND05 blocks stress-induced changes in many organs. Notably, the ability of CTRND05 to prevent CVS-induced weight loss and mesenteric fat accumulation has not been observed with reported pharmacologic manipulations of the HPA axis. Behavioral testing following CVS showed no significant differences in the elevated plus maze test or the forced-swim test, but there was a decrease in the fecal pellets produced during the forced swim test in the CTRND05 treatment groups, potentially indicating a decrease in stress-induced bowel motility (Fig. S2, P–R).

**Figure 3.**
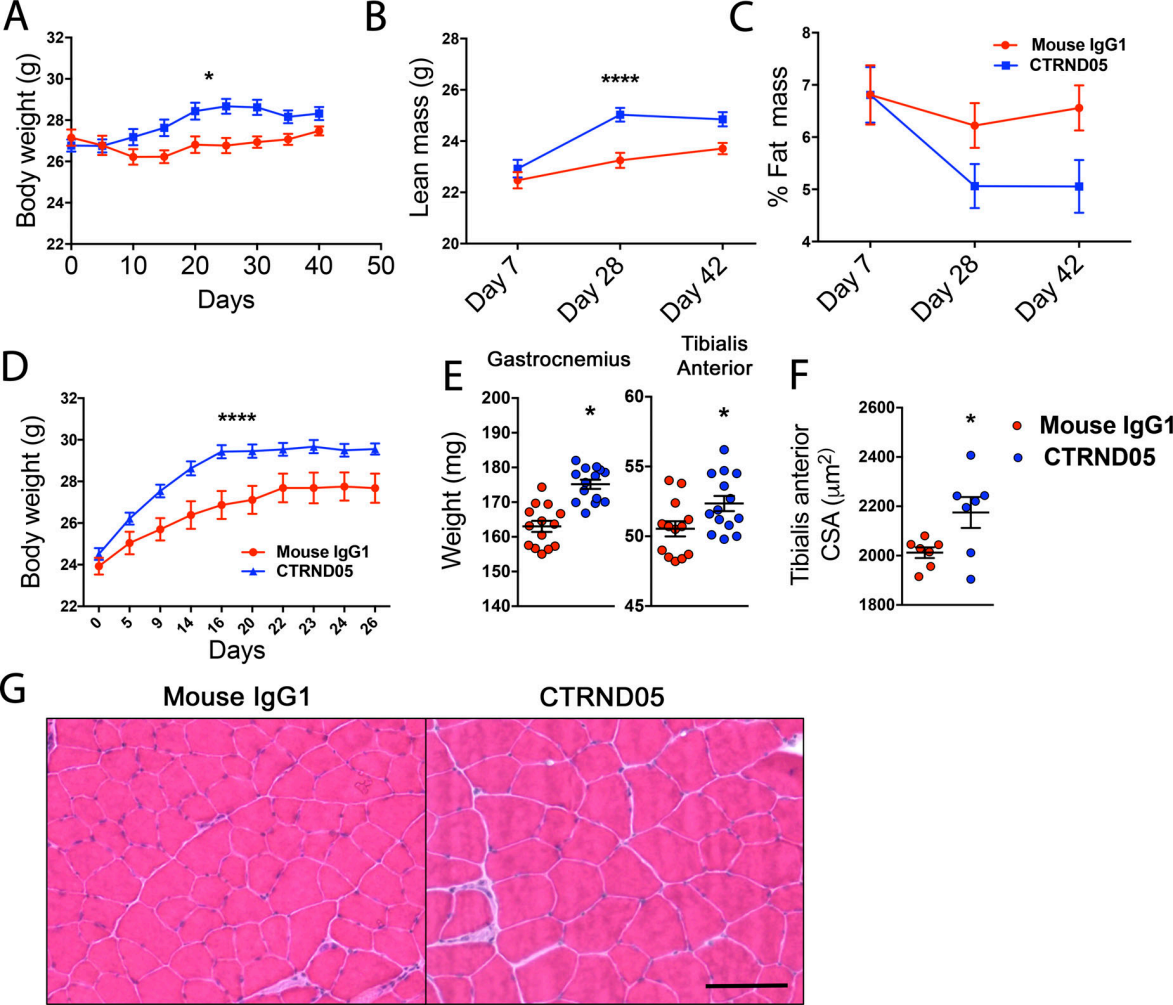
**CTRND05 treatment induces lean mass gain and skeletal muscle hypertrophy**. **(A–C)** NTg C57BL/6J mice (*n* = 8 males from one independent experiment) were individually housed and treated with CTRND05 or mouse IgG1 as above for 6 wk. Mice underwent continuous metabolic monitoring (days 1–7 and 21). Body composition was evaluated via EchoMRI (days 7, 28, and 42). **(A)** Treated mice gained significantly more weight over the course of 2–3 wk of treatment, and weight then plateaued. **(B and C)** CTRND05-treated mice gained lean mass (B), with a trending decrease in body fat (C). **(D–G)** NTg CD2F1 mice (*n* = 7 males from one independent experiment) were treated with CTRND05 or mouse IgG1 as above for 26 d. **(D)** Body weight significantly increased with CTRND05 treatment and plateaued by day 20. **(E–G)** Gastrocnemius and TA muscle weights significantly increased following CTRND05 treatment (E), and cross-sectional analysis demonstrates significantly increased myofiber cross-sectional area (F and G; scale bar = 100 µM). Error bars are represented as mean ± SEM. Statistics: two-way ANOVA (A, B, and D); unpaired *t* test (E and F); *, P < 0.05; ****, P < 0.0001.

To further investigate the observed weight changes induced by CTRND05, C57BL/6J NTg mice (*n* = 8 males) were singly housed and treated with CTRND05 for 6 wk with no additional stress paradigm. Mice underwent continuous metabolic monitoring on days 1–7 and day 21. On days 7, 28, and 42, body composition of the mice was evaluated via EchoMRI. In this paradigm, CTRND05 treatment also increased weight (Fig. 3 A), and EchoMRI demonstrated a gain of lean mass, and not an increase in fat mass or free water (Fig. 3, B and C; and Fig. S3 A). No significant differences in food intake, respiratory exchange ratio, or maximal oxygen uptake (VO_2_ max) were observed (Fig. S3, B–H). Effects of CTRND05 on GC-responsive organs were again noted (Fig. S2, K–O). In another study, CD2F1 NTg mice (*n* = 7 males) were group-housed and treated with CTRND05 for 26 d. We again observed weight gain in the CTRND05 treatment group that plateaued after 20 d of treatment (Fig. 3 D). Tibialis anterior (TA) and gastrocnemius muscles taken from these mice displayed a significant increase in weight (Fig. 3 E), and cross-sectional analysis demonstrated myofiber hypertrophy (Fig. 3, F and G). Excess GCs can cause muscle atrophy, and skeletal muscle tissue–specific GR knockout mice experience muscular hypertrophy (Braun and Marks, 2015; Shimizu et al., 2015), but such effects have not been reported with a pharmacologic agent targeting the HPA axis.

**Figure 4.**
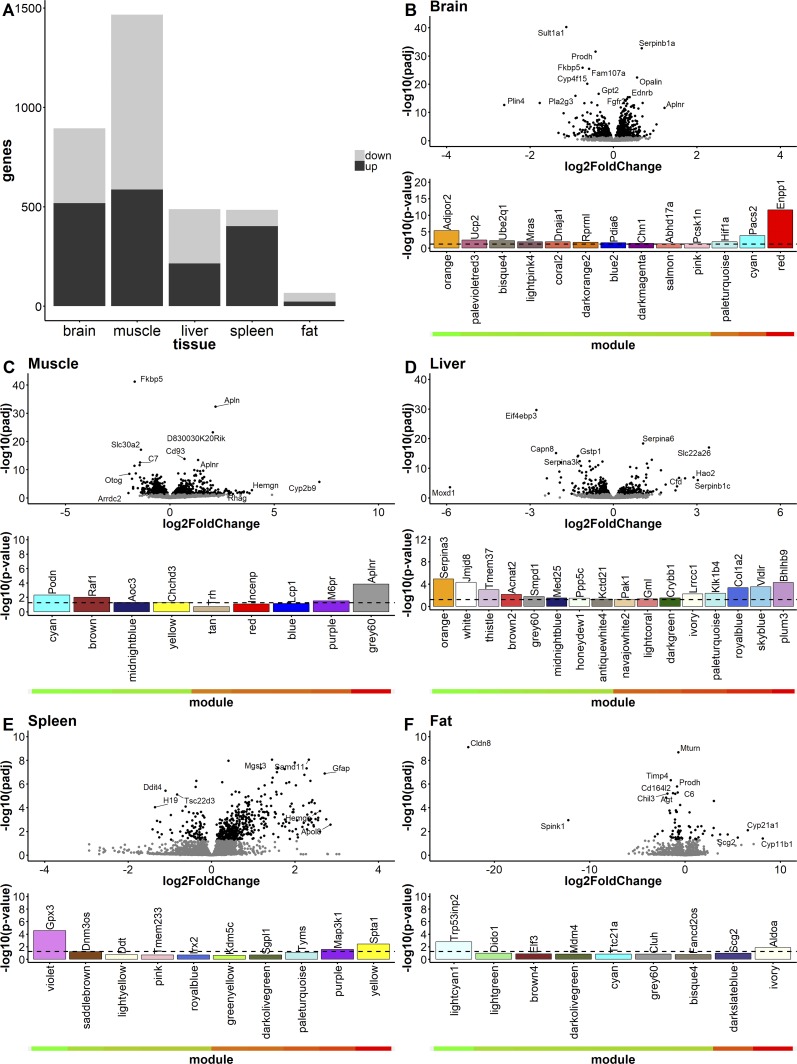
**CTRND05 treatment induces differential expression of hundreds of transcripts in five organs.** RNA transcriptomics of five organs from CTRND05-treated mice. **(A)** Number of DEGs (filtered by adjusted P value [Padj] < 0.05) in brain, muscle, liver, spleen, and fat. **(B–F)** Volcano plots for brain (B), muscle (C), liver (D), spleen (E), and fat tissue (F). Underneath, volcano plots of WGCNA modules are shown for relationship to antibody status. Genes with adjusted P values < 0.05 are indicated in black. Modules are graphed by significance of the module to antibody treatment (−log10(P value)) on the y axis and are arranged by correlation of the module to antibody treatment on the x axis with a heatmap demonstrating correlation values below (green, negative; red, positive). For ease in viewing, not all modules are shown (modules with P values above the following cutoffs are not shown: brain, 0.05; muscle, 0.2; liver, 0.05; spleen, 0.25; and fat, 0.2). Brains were harvested from mice in experiment shown in Fig. 2 (*n* = 12, 6 male/6 female). Muscle, liver, spleen, and fat were harvested from mice in experiment shown in Fig. 3 (A–C) (*n* = 6 male).

Given the pleiotropic phenotypic impact of blocking CRF and suppressing downstream HPA axis activation, we used a systems-level transcriptomic approach to evaluate effects on gene regulation in the brain, muscle, spleen, liver, and gonadal fat from mice treated with CTRND05 (Dataset 1). Though many previous studies have explored GC-responsive genes (Kuo et al., 2013; Gray et al., 2017), a recent review of these studies focusing on the brain identified just 88 transcripts that were consistently changed in response to GC (Juszczak and Stankiewicz, 2018). With the caveats that our study explores the effect of GC and CRF suppression by CTRND05 as opposed to excess GCs, the RNA sequencing (RNA-seq) data reveal large transcriptional changes in the brain and other organs (Fig. 4, A–F; and Dataset 1). Using a false discovery rate cutoff of 0.05, CTRND05 treatment significantly altered expression of 4.9% of the total gene transcripts detected in the brain (894 differentially expressed genes [DEGs]). In the muscle, liver, spleen, and fat, the percentages of DEGs were 8.3%, 3.1%, 2.7%, and 0.37%, respectively, and the absolute number of DEGs were 1,466 (muscle), 488 (liver), 484 (spleen), and 66 (fat; Fig. 4 A). Changes in gene expression in each organ were in select cases opposite of the reported transcriptional response to elevated GCs (Fig. 4, A–F). For example, *Fkbp5*, which is elevated following excess GCs, was down-regulated in all tissues examined, and statistically significant in four of five tissues (Dataset 1). However, most transcriptional changes were unique to each organ (Fig. 4, A–F; and Dataset 1). Weighted gene coexpression network analysis (WGCNA) of the data from each organ demonstrated hub genes as potential drivers of the observed network changes (Fig. 4, A–F; and Dataset 1). Collectively, these data provide a comprehensive assessment of transcriptional changes induced by subchronic HPA axis suppression and demonstrate the broad multiorgan impact at a gene regulatory level of HPA axis suppression by CTRND05. Such data can serve as an initial well-powered systems-level reference dataset for GC/CRF-responsive pathways in these organs. Notably, neither *Crh, Crhr1*, *Crhr2*, *Crhbp*, nor *Ucn1-3* transcript levels were significantly altered in the brain, liver, spleen, or fat; however, there was a significant down-regulation of *Crhr2* and *Ucn* (UCN1) transcripts in the skeletal muscle (Dataset 1).

Although specific transcript changes such as widespread down-regulation of *Fkbp5* and up-regulation of *SerpinA6* (Serpin Family A Member 6, corticosteroid-binding globulin) in the liver are predictable consequences of the subchronic GC suppression mediated by CTRND05 (Hammond, 2016), other specific and system-level gene expression changes observed in this multiorgan survey were quite unexpected. Of particular interest to our group are (i) the up-regulation of oligodendrocyte-specific transcripts related to myelination in the brain (Fig. 4 B and Dataset 1; Cheng et al., 2012), (ii) up-regulation of the growth factor apelin (*Apln*) and its receptor (*Aplnr*) in the brain and muscle (Fig. 4, B and C; and Dataset 1), and (iii) numerous DEGs and networks in the liver that link CTRND05 treatment to regulation of lipid metabolism (Fig. 4 D and Dataset 1). Previous studies have shown that GCs regulate oligodendrocyte and Schwann cell survival and function ex vivo. However, a direct link between suppressing GCs and CRF and oligodendrocyte function in vivo has not, to our knowledge, been demonstrated previously. Given the well-established negative impacts of excessive GCs on brain function and structure in Cushing’s syndrome and more recent data that associates high, but still physiological, cortisol levels with impaired memory and alterations in brain structure, especially in white matter tracts (Echouffo-Tcheugui et al., 2018), we find this current data intriguing. In the brain, apelin signaling has been implicated in promoting neuronal survival; thus, up-regulation of this signaling pathway could also potentially be neuroprotective. In muscle, not only are *Apln* and *Aplnr* transcripts increased, but WGCNA demonstrated that *Aplnr* is a hub gene (grey60 module; Fig. 4 C and Dataset 1). As apelin loss during aging was recently proposed to promote sarcopenia (Vinel et al., 2018), up-regulation of this signaling pathway, along with the observed decrease in transcripts of the myogenesis inhibitor *Myostatin* (*Mstn*), are strong candidates for mediating the muscle hypertrophy we observe following CTRND05 treatment. As *Mstn* is known to be GC responsive (Braun and Marks, 2015), this change could have been predicted, but the up-regulation of *Apln-Aplnr* was unexpected.

In the liver, gene ontology analysis of the DEGs and WGCNA-derived modules reveal extensive changes in genes that regulate lipid metabolism. For example, of the 488 DEGs in the liver, 67 are linked by gene ontology to lipid metabolism (Dataset 1). These changes include up-regulation of the very-low-density lipoprotein receptor (*Vldlr*), sortilin-related receptor (*Sorl1*), and long-chain fatty acid transport protein 1 (*Slc27a1*) and down-regulation of the microsomal triglyceride transfer protein large subunit (*Mttp*), mitochondrial sterol 26-hydroxylase (*Cyp27a1*) orthologue, and the steroid 17-α-hydroxylase (*Cyp17a1*). Given the longstanding links between hypercortisolism and metabolic syndrome (Moraitis et al., 2017), these data should provide a framework for studies that more thoroughly examine how GCs have diverse metabolic impacts.

Collectively, these data demonstrate that CTRND05 is capable of dose-dependently suppressing the HPA axis in vivo. Because of these actions, CTRND05 represents a valuable tool for selective pharmacologic suppression of the HPA axis, enabling both acute and chronic studies that explore the effects of varying degrees of HPA axis suppression in both physiological and pathological settings. Chronic psychological stress and GCs impact the immune system and glucose, lipid, and skeletal muscle metabolism in ways that are potentially deleterious to long-term health. Our preclinical studies demonstrate profound and rapid changes that occur with pharmacologic suppression of the HPA axis by CTRND05. Some of these changes, such as increases in lean body mass and skeletal muscle mass, were unexpected.

Although the current studies have mainly focused on the peripheral effects of CTRND05, we do see large-scale alterations in gene expression and down-regulation of the mRNA for the key GR chaperone FKBP5 in the brain, which is a target in major depression and may have other interesting chaperone functions relevant to tauopathy and neurodegeneration (Blair et al., 2013). Further, these brain transcriptomic studies reveal somewhat unexpected effects on oligodendrocyte gene expression and more predictable effects on immune gene expression in the brain. Overall, the multiorgan pharmacotranscriptomic studies show robust regulation of numerous genes and pathways that likely mediate some of the observed physiological effects of CTRND05. These studies reveal both novel and expected effects on gene expression and provide a rich resource that can help guide investigation of downstream effects of HPA axis suppression. Indeed, the effects of CTRND05 on lean body mass, muscle hypertrophy, oligodendrocyte transcriptional profiles, and cellular and transcriptional immune profiles all point to novel biological connections between the HPA axis, CRF-mediated signaling pathways, and these in vivo effects. Given the diversity of the DEGs and the WGCNA-derived transcriptional networks observed from organ to organ, these transcriptomic data can be mined to provide insight into GC/CRF-responsive pathways. In the future, transcriptomic studies from mice exposed to chronic CRF/GC excess (e.g., in a Cushingoid mouse model) can provide a complementary dataset, enabling rigorous comparisons of transcriptional changes that occur when the HPA axis is suppressed versus when it is overactive.

As CRF acts within the hypophyseal portal to stimulate ACTH production, our data show clear evidence for target engagement at this site, which is considered open to the peripheral circulation. CRFR1 receptors are distributed throughout the brain, and CRF has been shown to have direct actions within the brain (Hauger et al., 2006). Despite the large-scale alterations in brain transcriptomics induced by CTRND05 treatment, it is not clear from our current studies whether we are also blocking CRF actions in the CNS or whether these transcriptomic changes simply reflect suppression of GC signaling. High doses of CTRND05 (e.g., 25 mg/kg), which achieve ∼1–2-µM concentrations in blood, should result in ∼1-nM levels of the antibody in the CNS, as the CNS penetrance of most monoclonal antibodies is ∼0.1% of blood levels (Levites et al., 2006; St-Amour et al., 2013). Theoretically, this concentration of CTRND05 could engage CRF present at low picomolar levels in the brain. However, further studies evaluating this assertion directly are needed to establish if CTRND05 engages CNS CRF at sufficient levels to have functional impacts on brain CRF signaling pathways.

There have been many challenges (e.g., potency, specificity, toxicity, and GPCR vs. β-arrestin signaling) of targeting the CRF–CRFR1 axis and, more generally, neuropeptide-based signaling pathways with small molecules. Thus, given the emergence of recombinant antibody–based therapies targeting many different proteins for many different human disorders, it is somewhat surprising that there have not been more reports describing efforts to develop immunotherapies for CNS peptides that have impact on behavioral and metabolic pathways. Previous reports of immunoneutralization of CRF using polyclonal antisera and nanomolar-affinity monoclonals exhibited varying degrees of HPA axis suppression; however, those studies were quite limited and not translatable (Linton et al., 1985; Sapolsky et al., 1987; Giuffre et al., 1988; van Oers et al., 1989; Milton et al., 1990; Potter et al., 1992; Pich et al., 1993; Kravchenko and Furalev, 1994). Our current studies clearly demonstrate that the affinity of the anti-CRF antibody is important, as neither lower-affinity (nanomolar K_d_) anti-CRF antibodies nor active immunotherapy that induces a high-titer anti-CRF response blocked HPA axis activation. Indeed, given the low-picomolar levels of CRF normally present, monoclonal antibodies require very high affinity to obtain dramatic and sustained HPA axis suppression, as we have demonstrated in these studies. As many neuropeptides are present at low concentrations, passive immunotherapy targeting CRF is also an exemplar, such as anti-CGRP antibodies for migraine (Giamberardino et al., 2016), that may anticipate future studies in which high-affinity antibodies targeting a broad range of neuropeptides are used as both research tools and possible therapeutics.

The CRF protein is completely conserved between humans and mice and is present at similar concentrations (Chang and Hsu, 2004). These studies provide the initial rationale for development of a humanized high-affinity anti-CRF antibody. Clearly, given the potential for some interaction with UCN1, albeit at lower binding affinity, future humanization efforts must take potential UCN1 binding into consideration, and additional studies will need to be conducted to determine the extent of UCN1 engagement in vivo. Such a therapeutic would enable the definitive testing of the proposed health benefits of suppressing the HPA axis in a plethora of human diseases and disorders.

## Materials and methods

### Mouse strains and housing

B6C3H-F1 (#061) and C57BL/6J (#044) mice were obtained from Envigo. All animal procedures were performed with approval from the University of Florida Institutional Animal Care and Use Committee. BALB/c (#028) and CDF1 (#033) mice were obtained from Charles River. Depending on experiment, mice were housed either singly or in groups (see figure legends). All mice were NTg strains, and all experiments used age-matched mice at 8–12 wk of age when initiating the study. Mice were given ad libitum access to pelleted rodent food and water on a 12-h/12-h light-dark cycle. The light phase started at 0700 and the dark phase started at 1900.

### Immunization for inducing anti-CRF titers in mice

Antibodies were developed by immunizing NTg BALB/c mice against CRF using immunogens as described previously (Croft et al., 2018). For immunization, two sections of CRF were conjugated to keyhole limpet hemocyanin (KLH) and OVA (residues shown in Fig. S1, A and B). Peptides were synthesized by Genscript. 200 µg of immunogen was injected into NTg mice in CFA (#F5881; Sigma-Aldrich) and boosted twice at 2-wk intervals with 200 µg of immunogen in incomplete Freund’s adjuvant (#F5506; Sigma-Aldrich). Mice were then bled via mandibular vein, and serum anti-CRF titers were checked via direct ELISA.

### ELISA for antibody titer

For detection of anti-CRF antibodies, CRF-KLH or CRF-OVA from above was coated to Immulon clear 4HBX 96-well plates (#14245153; Thermo Fisher) at 1 µg/ml in 100 mM NaCO_3_ overnight at 4°C. Plates were washed with PBS twice and blocked with Block Ace solution for 3 h at room temperature. Plates were washed with PBS twice, and sera were diluted in phosphate-based assay buffer and incubated on the plate for 2 h at room temperature. Plates were washed again with PBS twice, and goat anti-mouse secondary antibody conjugated to HRP (#115-035-003; Jackson ImmunoResearch Labs) secondary antibody was diluted (1:5,000) in buffer and incubated on the plate for 1 h. Plates were then washed with PBST twice followed by PBS twice, and TMB substrates (#5067422; Pierce) were applied for 10 min. Reactions were quenched with 1 M HCl or 85% *O*-phosphoric acid, and absorbance was measured at 450 nm using a Clariostar spectrophotometer from BMG Labtech.

### Generation of hybridoma-producing anti-CRF antibodies

Spleens were harvested from mice with strong anti-CRF titers. Spleens were gently homogenized in 5% FBS/HBSS from Lonza and centrifuged to pellet cells. The cell pellet was resuspended in red blood cell lysis buffer from Sigma-Aldrich and diluted with HBSS after 1 min. The cells were washed twice by centrifuging at 100 *g* for 10 min and resuspended in HBSS. Sp2/O-Ag14 cells (ATCC) were also washed twice with HBSS. Five million Sp2/O-Ag14 cells were added to 50 million spleen cells and, after centrifuging at 100 *g* for 10 min onto a culture dish, fusion was induced with 50% polyethylene glycol 1450 (PEG; Sigma-Aldrich). After washing with HBSS, cells were incubated in Sp2/O-Ag14 medium at 37°C with 8% CO_2_ overnight. The next day, the cells were gently detached from the plate and distributed into 96-well plates with Sp2/O-Ag14 medium/0.5% hybridoma-enhancing supplement from Sigma-Aldrich. After 1 wk, hybridomas were tested for anti-CRF titers via direct ELISA described above, and positive clones were expanded. These positive polyclonal hybridoma lines were then frozen at −150°C and subsequently subcloned.

### Control antibody

The control mouse IgG1 antibody used in all studies was an IgG1 antibody produced by our center via murine hybridomas as above. The antibody is specific for the N-terminal domain of human amyloid-β peptide that does not bind a target in NTg mice (Kim et al., 2007).

### Antibody purification

Antibodies were produced by collecting ascites fluid from hybridomas grown in mice. Mouse IgG1 was purified using Protein A columns. This was contracted work from QED Biosciences.

### Antibody isotyping

Mouse antibodies were isotyped according to instructions using a Thermo Fisher Mouse Isotyping ELISA kit (#37503).

### Biotinylation of CTRND05, CRF, and UCN peptides

PEG4 biotinylating reagent (#21330; Thermo Fisher) was used at a 20:1 molar ratio to biotinylate CTRND05 antibody, CRF peptide, or UCN1-3 peptides (#4011473, #4027201, #4040984, #4039202; Bachem). CRF-biotin and UCN1-3-biotin was then desalted using a 2-kD dialysis membrane (#G235035; Spectrum Labs) in PBS for 20 h. CTRND05-biotin was desalted using desalting columns (Zeba Spin #89892; Thermo Fisher).

### CRF-CTRND05 and UCN1-3 competitive ELISA

CRF from Bachem was coated to Immulon clear 4HBX 96-well plates (#14245153; Thermo Fisher) at 10 nM and 1,000 nM in 100 mM NaCO_3_ overnight at 4°C. Plates were then washed with PBS twice and blocked with Block Ace solution for 3 h at room temperature. Plates were washed with PBS twice, and solutions containing final concentrations of 20 nM CTRND05 and varying concentrations of UCN1, UCN2, or UCN3 were placed onto the plate and incubated for 2 h at room temperature. Plates were washed again with PBS twice, and goat anti-mouse secondary antibody conjugated to HRP (#115-035-003; Jackson ImmunoResearch Labs) secondary antibody was diluted (1:5,000) in buffer and incubated on the plate for 1 h. Plates were washed with PBS with Tween twice followed by PBS twice, and TMB substrates (#5067422; Pierce) were applied for 10 min. Reactions were then quenched with 1 M HCl or 85% *O*-phosphoric acid, and absorbance was measured at 450 nm using the Clariostar spectrophotometer from BMG Labtech.

### Octet-red BLI to determine antibody affinity

BLI was used for K_d_ (equilibrium dissociation constant) determination using Streptavidin Biosensors on the Octet Red384 platform (FortéBio; Pall Life Sciences). CRF and UCN1-3 peptides were biotinylated with Pierce EZ-Link NHS-PEG4 (#21455; Thermo Fisher). Streptavidin Biosensors were equilibrated and loaded to near-saturation with biotin-CRF in PBS (assay buffer), transferred to fresh assay buffer for baseline measurement, then associated with monoclonal antibody CTRND05 as ligand along a serial dilution. The sensors were finally moved back to assay buffer for disassociation. On rate (K_on_), off rate (K_off_), and K_d_ (K_on_/K_off_) values were determined by global fitting of the binding curves for the ligand dilutions and calculated by applying a 1:1 interaction model using the FortéBio Data Analysis software version 9.0.0.14 (FortéBio; Pall Life Sciences).

### H4 CRFR1-GFP stable cells

Stable CRFR1-GFP overexpressing H4 neuroglioma cells were generated as previously described (Park et al., 2015). Briefly, CRFR1-GFP fusion transgene with zeocin selection transgene was generated via molecular cloning methods and restriction enzyme digestion. H4 neuroglioma cells were transfected using Fugene transfection reagent and were placed into Zeocin (R25005; Thermo Fisher) at 200 µg/ml in OptiMEM in 6% FBS. After 1 wk on selection, cells were split to a 96-well plate at 1 cell/well, and clonal expressing populations were then identified over the next 2–3 wk and expanded and frozen in 95% FBS and 5% DMSO.

### cAMP measurement

H4 cells stably overexpressing CRFR1-GFP were plated in a 96-well plate and treated with varying concentrations of CRF peptide (#4011473; Bachem) solubilized in DMSO, in addition to CTRND05 in PBS for 30 min. Cells were then lysed, and cAMP was measured using an Invitrogen competitive ELISA kit (#EMSCAMPL) according to kit instructions.

### rAAV vector creation and virus production and titer

Restriction endonuclease molecular cloning methods were used to insert the mouse CRF transgene (synthesized by Genscript) into an rAAV IRES-GFP vector. Virus purification and titer were as described in Chakrabarty et al. (2013). HEK293t cells were then cotransfected with this CRF-IRES-GFP vector, in addition to pHelper for rAAV8 from Plasmid Factory using polyethylenimine (#NC1501492; Sigma-Aldrich). Cells were incubated for 72 h, and cells were lysed and centrifuged in an Iodixanol density gradient to allow for purification of pure rAAV capsids. Virus was used at 1.0 × 10^13^ vector genomes/ml. An rAAV2/8 IRES-GFP vector with no CRF at the same titer was used as a control.

### Postnatal day 0 (P0) intracerebroventricular injection

Injections were performed as described in Chakrabarty et al. (2013). Briefly, P0 pups were cryoanesthetized in aluminum foil on wet ice for 5 min. 2 ml containing 1.0 × 10^13^ purified AAV8 was injected bilaterally into the lateral cerebral ventricles for a total of 4 µl per animal, using a Hamilton 800 series 10 µl syringe (#20797; Sigma-Aldrich). Pups were then rewarmed and then placed back in the mother’s cage.

### Immune profiling

Spleens were processed with frosted glass slides and filtered (70 µm) to create single-cell suspensions. Red blood cells were lysed with ammonium-chloride-potassium lysis buffer for 5 min on ice, and remaining cells were washed with PBS before staining. 10^6^ cells per sample were stained with Fixable Live/Dead Near Infrared (#L34975; Thermo Fisher) for dead cell exclusion. Cells were incubated with Fc block (2.4G2; BD Biosciences) for 5 min on ice before staining with the following antibodies at appropriate concentrations for 30 min on ice: CD4-PerCP-Cy5.5 (RM4-5; eBioscience), CD8a-PE-Cy7 (53-6.7; BioLegend), CD3e-BV605 (145-2C11; BioLegend), NK1.1-APC (PK136; eBioscience), CD19-BV711 (6D5; BioLegend), Ly6G-BV421 (1A8; BD Biosciences), Ly6C-PE (HK1.4; eBioscience), and CD11b-AF488 (M1/70; eBioscience). Samples were washed once before data acquisition on an LSR Fortessa (BD Biosciences) and analysis using FlowJo (v10.5.0; TreeStar).

### Restraint stress

Mice were placed into Broome-style rodent restrainers (#551-BSRR; Plas-Labs) for 30 min, with the restrainer being kept in the mouse’s home cage during this time. All stress experiments were performed from 0600 to 0900.

### Plasma draw

All plasma draws for basal corticosterone measurement were performed within 2 min of touching the cage. A small (∼1-mm) snip of the tip of the mouse tail was removed, and two to three drops of blood (∼20–30 µl) were milked gently into a capillary plasma collection tube (Microvette CB300 K2E) from Sarstedt. For multi–time point experiments, samples of this size were milked out of the mouse tail at each time point (30, 90, and 150 min). Tubes were kept on ice and then centrifuged at 4,500 relative centrifugal force for 15 min. Supernatant plasma was then collected and stored at −80°C until use. For baseline corticosterone measurement, plasma was collected from 0700 to 0800.

### Corticosterone measurement

MP Bio corticosterone double antibody RIA Kit (07120102) was used according to instructions. Mouse plasma was diluted 1:100 in assay buffer for nonstress basal conditions, 1:200 for recovery time points, and 1:400 for maximum-stress time points.

### Metabolic monitoring

Energy expenditure and food intake were measured using an automated indirect calorimetric system, PhenoMaster, that is situated in a climate-controlled chamber from TSE Systems. The environmental conditions in the chamber were set to 25°C, 50% humidity, and a 12-h/12-h light/dark cycle, and mice were adapted to the system for 24 h before experimental data were collected. Oxygen consumption and CO_2_ production were measured every 15 min for the duration of the studies. These values were then used to calculate the respiratory quotient (VCO_2_/VO_2_) and energy expenditure using PhenoMaster software. Food intake was determined continuously by scales that are integrated into the PhenoMaster System.

### EchoMRI

Body composition was quantified in conscious mice on days 7, 28, and 42 after treatment using EchoMRI Quantitative Magnetic Resonance Body Composition Analyzer (Echo Medical Systems).

### CVS

The chronic stress protocol consisted of twice-daily (morning and afternoon) exposure to randomly assigned stressors for 2 wk. Other than to record weight and food intake, control animals were not disturbed. Morning stressors were conducted between 0800 and 1130, and afternoon stressors were administered between 1330 and 1700. Stressors consisted of rotation stress (1 h at 100 rpm on a platform orbital shaker), cold room stress (kept in 10°C for 1 h), hypoxia (8% O_2_ and 92% N_2_), and being placed into a novel cage for 10 min.

### Elevated plus maze

Anxiety-like behavior was assessed during the light phase (0800–1200) using an elevated plus maze that consists of two opposing closed arms and two opposing open arms (31 × 6 cm for each arm) elevated 41 cm above the floor. On the day of the test, mice were brought into the testing room and again habituated to the tether in their home cage for 5 min. Mice were then placed in the center of the elevated plus maze and allowed to explore the maze for a period of 5 min.

### Forced swim test

Female and male mice were placed in a 2-liter beaker half-filled with water (23 ± 2°C). This level of water prevents the mice from reaching the bottom of the container and from escaping. Each session was videotaped, and all behaviors were scored in 5-s intervals by two independent observers who were blind to the genotype or sex of each mouse. The total count of each behavior during the 10-min testing session was summed for each animal and averaged based on the group. The behaviors scored are defined as follows: (i) climbing, rapid movement of limbs in and out of the water with the body parallel to the apparatus; (ii) diving; (iii) swimming, moving limbs in an active manner and making circular movements around the apparatus; and immobility, no active movements or floating in the water without struggling.

### Organ harvest

At study endpoint, mice underwent decapitation and had organs dissected, rinsed in PBS, and weighed on a scale (#AL54; Mettler Toledo). For liver harvest, the left lobe of the liver was harvested from each mouse.

### Muscle harvest and measurement of muscle fiber cross-sectional area

At study endpoint, mice were anesthetized via isoflurane for muscle harvest. The TA and gastrocnemius complex (gastrocnemius, plantaris, and soleus) were harvested from the hindlimbs and weighed immediately. The TA muscles were embedded in optimum cutting temperature medium and frozen in liquid nitrogen–chilled isopentane for histological analysis. On a microtome cryostat, 10-µm-thick cross sections of the TA muscles were cut and transferred to glass slides for staining with H&E. Tissue sections were imaged on a Leica DM5000 B upright microscope (Wetzlar) equipped with a 32-bit DFC425 color camera, and images were captured using a 10×/0.3 objective. Data were acquired with LAS Core software (Leica Microsystems). Quantification of muscle fiber cross-sectional area was performed by importing TIF files of H&E-stained sections into ImageJ, and then hand-tracing and quantifying the areas of individual muscle fibers. Cross-sectional area measurements were collected from two transverse sections of the midbelly of the TA muscle separated by 50 µm. Seven muscles were analyzed per group.

### RNA-seq and analysis

Brains were harvested from mice in the experiment shown in Fig. 2 (A–F) (*n* = 6, 3 male/3 female B6C3H-F1 mice). Muscle, liver, spleen, and fat were harvested from mice in the experiment shown in Fig. 3 (A–C) (*n* = 6 male C57BL/6J mice). Harvested organs were snap-frozen in isopentane chilled on dry ice. CTRND05-treated and control tissues were pulverized under liquid nitrogen using a mortar and pestle, and total RNA was extracted with Trizol reagent (#15596026; Thermo Fisher). RNA was cleaned up over a Qiagen RNeasy column with on-column DNase treatment. A subsequent DNase treatment using Turbo DNA-free kit (AM1907; Thermo Fisher) was performed. RNA was quantified using the Qubit 4 fluorometer and the RNA HS assay (Q32852; Thermo Fisher). RNA quality was determined using the Fragment Analyzer Automated CE System and the Standard Sensitivity RNA Analysis kit (Agilent). 1 µg of total RNA was polyA enriched and subjected to library preparation using the TruSeq RNA Sample Prep Kit v2 (RS-122-2001; Illumina). Libraries were quantified using a library quantification kit (KAPA Biosystems). Library size was determined with the High-Sensitivity NGS Fragment Analysis Kit (Agilent). Libraries were pooled with a strategy to minimize batch effects from library preparation and sequencing and to achieve 30–50 million reads per sample.

The resulting FASTQ files were aligned against the mouse genome using STAR (Dobin et al., 2013). Subsequent analyses were performed in R version 3.5.1. Differential gene expression analysis was performed with DESeq2. Changes in gene expression levels between treated versus control groups within each tissue were compared with “find DEGs.” WGCNA was performed to determine modules of genes with similar expression patterns across samples (Langfelder and Horvath, 2007, 2008). Signed hybrid networks were detected with WGCNA using soft power settings (β) of 6, 4, 6, 4, and 7 for brain, muscle, liver, spleen, and fat tissues, respectively. Identification of intramodular hub genes from modules that were significantly correlated with the antibody treatment group were examined, and network pathways and gene ontology of genes within significant modules were analyzed using anRichment associated with the WGCNA package. Networks were visualized using the geomnet package in R using the top 20% of genes belonging to each module.

### Oligodendrocyte module comparison

The RNA-seq data derived from Fig. 4 B were analyzed, and oligodendrocyte related transcripts were identified as defined in Cahoy et al. (2008) and Zhang et al. (2014). We then compared the number of transcripts from this collection of transcripts between treatment groups by determining the geometric mean of the fragments per kilobase of transcript per million mapped reads. Plots (Dataset 1) were made with ggplot2 in R.

### Statistical analysis

Data were analyzed using Prism 6 (GraphPad) and presented as mean ± SEM. Specific tests used are noted in the figure legends. Overall data were tested for normality and, after being deemed to have a normal distribution, were analyzed via Student’s *t* test, one-way ANOVA followed by Dunnett’s multiple comparison test, two-way ANOVA, or Dunn’s test.

### Data and materials availability

All transcriptomic DEG data, WGCNA modules, networks, and code are uploaded on Synapse (https://www.synapse.org/) and available for free use with project ID syn18796452.

### Online supplemental material

Fig. S1 displays pharmacokinetic and characterizing studies for CTRND05. Fig. S2 displays effects of CTRND05 on GC-sensitive organs and mouse behavior. Fig S3 displays effects of CTRND05 on energy expenditure and food intake. Dataset 1 provides lists of DEGs, DEG overlap, and WGCNA modules.

## Supplementary Material

Supplemental Materials (PDF)

Dataset 1 (Excel file)
