# Supplementary material for: An anti-CRF antibody suppresses the HPA axis and reverses stress-induced phenotypes
Source: J Exp Med. 2019 Aug 29;216(11):2479–91. doi: 10.1084/jem.20190430 (PMC6829597; doi:10.1084/jem.20190430)
Supplement: Supplemental Materials (PDF) [file JEM_20190430_sm.pdf]

Supplemental material

Futch et al., https://doi.org/10.1084/jem.20190430

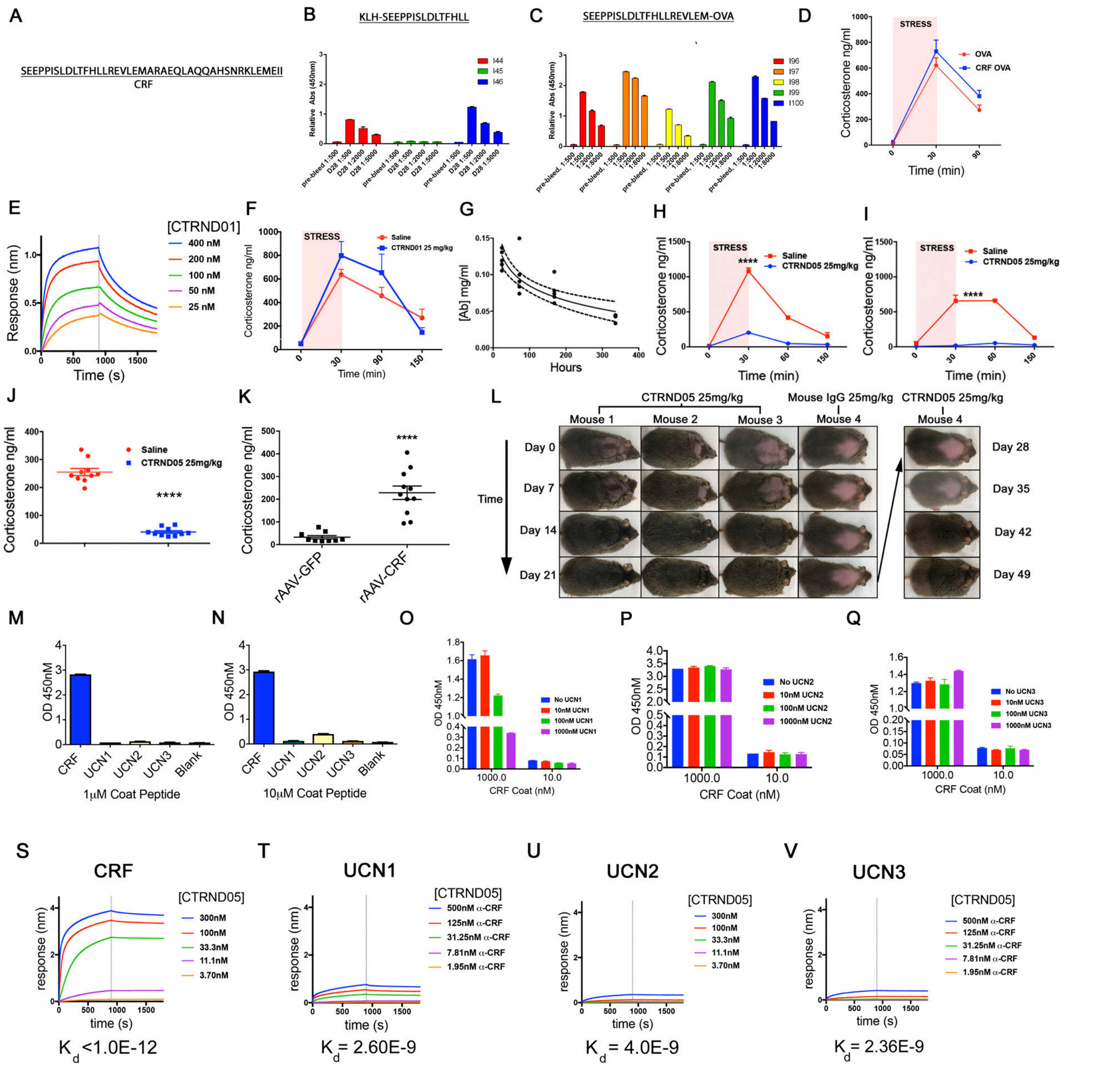

Figure S1. **Generation and characterization of anti-CRF antibodies. (A)** Sequence of human/mouse/rat CRF. **(B and C)** Serum titers against CRF as detected via direct ELISA. In B, *n* = 3 female Balb/c mice from one independent experiment, and in C, *n* = 5 female Balb/c mice from one independent experiment, immunized with CRF peptides conjugated to either KLH (B) or OVA (C). The longer OVA-conjugated peptide (C) generated consistent high titers. **(D)** B6C3H-F1 NTg mice (*n* = 8, 4 male/4 female, from one independent experiment) were vaccinated with the CRF-OVA vaccine that was used to generate CTRND05 on day 0 and boosted on days 14 and 28; no effect on corticosterone response was seen to 30-min restraint stress on day 30. **(E)** CTRND01 monoclonal antibody isolated from mice immunized with the KLH vaccine has a nanomolar affinity, with $K_d = 2.09 \times 10^{-8}$ (one independent experiment). **(F)** B6C3H-F1 NTg mice (*n* = 8, 4 male/4 female, from one independent experiment) injected i.p. with CTRND01 25 mg/kg 12–16 h before 30-min restraint stress had no appreciable effect on corticosterone response. **(G)** B6C3H-F1 mice (*n* = 5, 2 male/3 female, from one independent experiment) were injected with biotinylated CTRND05 and bled at 1 d, 3 d, 1 wk, and 2 wk. ELISA (incubating plasma on CRF-coated plates and detection with Streptavidin HRP) revealed the half-life of CTRND05 to be 1 wk. **(H–J)** Data from experiment in Fig. 1 E: B6C3H-F1 mice (*n* = 10, 5 male/5 female, from one independent experiment) injected with CTRND05 at 25 mg/kg 12–16 h before restraint stress. **(H)** Female mice display 80% blockade of 30-min restraint stress–induced rise in plasma corticosterone after treatment with 25 mg/kg CTRND05. **(I)** Male mice display >95% blockade under the same conditions. **(J)** Mice bled 30 min after handling at 1100 on day 5 after injection with CTRND05 remain at suppressed corticosterone levels. **(K)** B6C3H-F1 NTg (n = 4, 2 male/2 female, from one independent experiment) injected on P0 with 2 µl of 1.0×10^{13} vector genomes/ml rAAV2/8-CRF-IRES-GFP virus have markedly increased baseline plasma corticosterone levels. **(L)** rAAV-CRF–transduced cushingoid mice at 4 mo of age have hair loss reversed by a single i.p. dose of CTRND05 at 25 mg/kg (rightmost two columns are duplicates of those shown in Fig. 1). **(M and N)** Direct ELISA with CTRND05 binding to plate-bound CRF or UCN 1–3 revealed a small amount of signal for UCN2 when UCN2 was coated at a concentration of 10 µM but not at 1 µM (data from duplicate wells from one independent experiment). **(O–Q)** In a competitive ELISA where soluble UCNs compete with plate-bound CRF for CTRND05 binding, we observe that UCN1 displays a competitive effect at concentrations of 10 and 100 nM, while UCN2 and UNC3 show no competitive effect (data from two independent experiments). **(S–V)** Affinity measurement of CTRND05 to CRF (S) and UCNs (T–V) determined by Octet-Red BLI from two independent experiments. Error bars are represented as mean ± SEM. Statistics: unpaired *t* test (J and K); ****, P < 0.0001.

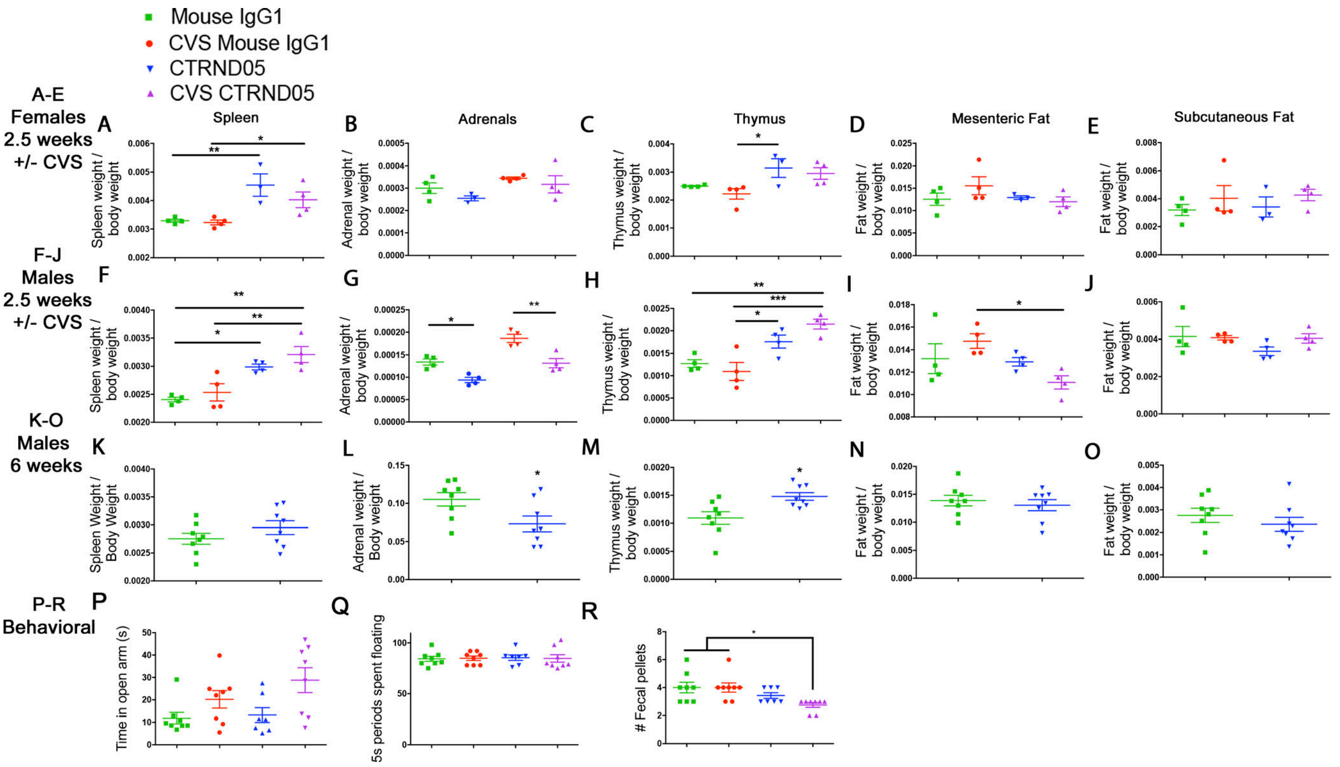

Figure S2. **GC-responsive organ weights and behavioral testing. (A–J and P–R)** Extended data from Fig. 2. CTRND05 blocks effects of CVS. **(K–O)** Extended data from Fig. 3. CTRND05 treatment induces lean mass gain and skeletal muscle hypertrophy. **(A–J)** GC-responsive organs taken from female (A–E) and male (F–J) C57BL/6J mice treated with CTRND05 for 2 wk with and without exposure to CVS display classic signs of HPA axis activation in CVS groups and blockade in CTRND05 groups. **(K–O)** Data from experiment in Fig. 3 (H and I) (n = 8 male C57BL/6J treated with CTRND05 for 6 wk) display shifts in GC responsive organ weights. **(P–R)** Behavioral testing performed on mice from experiment in Fig. 2 (A–F): time in open arm for mice in a 5-min elevated plus maze test for anxiety-like behavior (P) and number of 5-s periods spent floating in a 10-min forced swim test for depressive-like behavior (Q). **(R)** Numbers of fecal pellets during the forced swim test were decreased with CTRND05 treatment. Error bars are represented as mean ± SEM. Statistics: unpaired t test (J and K); *, P < 0.05; **, P < 0.01; ***, P < 0.001.

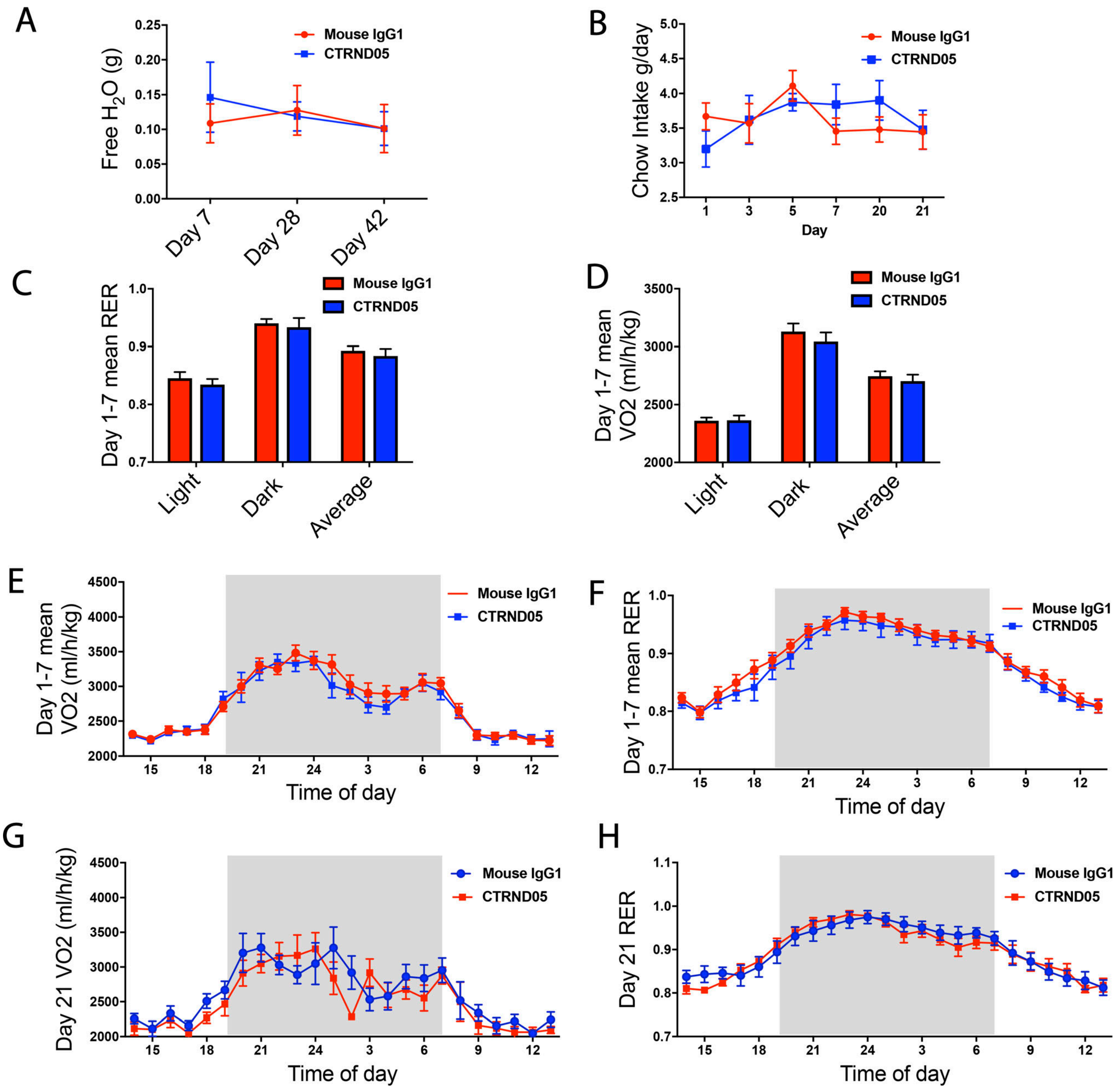

Figure S3. **Metabolic monitoring.** Extended data from Fig. 3. CTRND05 treatment induces lean mass gain and skeletal muscle hypertrophy. **(A)** Free H_2O measurements from EchoMRI measurements. **(B–H)** Metabolic monitoring of eight male C57BL/6J mice from experiment in Fig. 3 (A–C) reveals no differences in free H_2O, average VO_2, respiratory exchange ratio (RER), or chow intake on days 1–7 or day 21 after treatment with CTRND05, despite these mice having significantly increased body weight gain and lean mass. Error bars are represented as mean ± SEM.

Dataset 1 is provided online as a separate Excel file and provides lists of DEGs, DEG overlap, and WGCNA modules. Statistical analyses and methods can be found in the Materials and methods.
